# EHMTI-0099. Migraineur perception regarding family burden from chronic migraine: results of the CAMEO (chronic migraine epidemiology & outcomes) study

**DOI:** 10.1186/1129-2377-15-S1-D17

**Published:** 2014-09-18

**Authors:** DC Buse, DW Dodick, AM Adams

**Affiliations:** 1 The Saul R. Korey Department of Neurology, Albert Einstein College of Medicine and Montefiore Headache Center, Bronx, USA; 2 Neurology, Mayo Clinic, Phoenix, USA; 3 Global Medical Affairs, Allergan Inc., Irvine, USA

## Introduction

Chronic migraine (CM) is associated with personal disability and burden; however the effect on family life is less certain.

## Aims

The Family Burden Module (FBM) from the CaMEO study evaluated the extent, nature, and perceptions of burden of headache on migraineurs and their families.

## Methods

CaMEO recruited persons to participate in a series of web-based surveys over 1 year to characterize migraine. The panel surveyed was sociodemographically representative of the US. FBM-Proband survey assessed 15 domains (134 items), including missed activities with family, partner, and children, and relationship impact. Data from respondents meeting CM study criteria (modified International Classification of Headache Disorders, 3rd edition, beta [ICHD-3b] migraine diagnosis + ≥15 headache days/month for past 3 months; ICHD-3b criterion C not assessed) were included in current analysis.

## Results

11,518 respondents had valid data from the FBM; 994 (8.6%) probands were classified with CM. Probands reported reduced participation in family activities (6.9 days) and enjoyment of quality time with partners (6.6 days) within the preceding month. 20% reported missing vacations and reduced enjoyment at important family events (9.4 weeks) within the previous year. Most probands felt their headaches made their partner's (64.1%) and children's (56.5%) lives hard, and thought they would be better partners (72.5%) and parents (59.1%) without headaches.

## Conclusions

CM adversely affects family perception, relationships and activities. These findings suggest CM is a significant burden to migraineurs that extends to the family.

## Funding

Allergan.

